# Glucocorticoids and mineralocorticoids in hair: facilitating accurate diagnosis of adrenal-related endocrine disorders

**DOI:** 10.3389/fendo.2024.1448013

**Published:** 2024-11-14

**Authors:** Bianca Heyns, Rialet Pieters, Marietjie Aletta Stander, Stephen Lawrence Atkin, Amanda Cecilia Swart

**Affiliations:** ^1^ Department of Biochemistry, Stellenbosch University, Stellenbosch, South Africa; ^2^ Unit for Environmental Sciences and Management, North-West University, Potchefstroom, South Africa; ^3^ School Postgraduate Studies and Research, Royal College of Surgeons in Ireland, Adliya, Bahrain; ^4^ Department of Chemistry and Polymer Science, Stellenbosch University, Stellenbosch, South Africa

**Keywords:** hair cortisol, cortisone, aldosterone, chronic stress, aldosteronism, hair follicle, Cushing’s syndrome (CS), congenital adrenal hyperplasia (CAH)

## Abstract

**Background:**

Glucocorticoids and androgens in the hair follicle have been of interest for many years, particularly cortisol and cortisone in retrospective studies associated with chronic stress and Cushing’s syndrome. No studies have reported aldosterone or 18-hydroxycorticosterone in the adrenal mineralocorticoid panel in the hair follicle. This study aimed to identify potential biomarkers in endocrine conditions associated with steroid excess or deficiency using a novel extraction protocol in the analysis of mineralocorticoids and glucocorticoids in the hair follicle.

**Methods and findings:**

Hair was collected from 15 healthy male and female volunteers. Segments that were cut along the length of long, medium, and short hair and segments shaved on the scalp and the cheek were prepared for analysis. Hair samples were extracted using an automated accelerated solvent extraction (ASE) system. Steroids were analyzed using high-throughput ultra-performance convergence chromatography–tandem mass spectrometry. All mineralocorticoids and glucocorticoids were detected above the lower limit of quantification and none of the steroids differed statistically comparing male and female concentrations. Deoxycortisol, deoxycorticosterone, and aldosterone were detected for the first time in men. In both genders, 18-hydroxycortisosterone was detected for the first time. The median concentrations for women and men, respectively, were as follows: deoxycortisol, 14.2 and 19.2 pg/mg; cortisol, 34.7 and 33.9 pg/mg; cortisone, 22.4 and 22.0 pg/mg; deoxycorticosterone, 83.0 and 50.2 pg/mg; corticosterone, 10.9 and 11.5 pg/mg; 18-hydroxycorticosterone, 24.8 and 24.8 pg/mg; and aldosterone, 23.4 and 22.7 pg/mg. Deoxycortisol and deoxycorticosterone showed marked fluctuation along the hair follicle in both genders and showed inter-individual variance. Conversely, cortisol, cortisone, corticosterone, 18-hydroxycortisosterone, and aldosterone did not fluctuate, with no inter-individual variance. Cortisol was 1.5-fold higher than cortisone in accordance with the circulatory cortisol/cortisone relationship.

**Conclusions:**

The novel extraction method optimized steroid measurement, showing the consistency of measurement for glucocorticoids, cortisol and cortisone, and mineralocorticoids, corticosterone, 18-hydroxycorticosterone, and aldosterone. Data suggest these steroids in the hair follicle to be ideal biomarkers in improving diagnostic testing, investigating conditions of steroid excess or deficiency in hypoaldosteronism, primary hyperaldosteronism, Cushing’s syndrome, and the congenital adrenal hyperplasia subtypes.

## 1 Introduction

There has been increased interest in the analysis of steroids in matrices other than serum, plasma, and urine, in pursuit of their enhanced diagnostic accuracy for biomarkers associated with clinical conditions. These matrices include, among others, saliva, nails, and hair (1–5). Although analysis of steroids in hair follicles (hair follicle being defined as the entire hair length) has received a lot of attention, the primary focus has been predominantly on cortisol, testosterone (T), and dihydrotestosterone (DHT), with these steroids being linked to stress and hair loss. Steroids in the hair follicle reflect the long-term measure of the free steroids taken up from the bloodstream through constant passive diffusion. Levels are thus not confounded by diurnal fluctuations, allowing cumulative retrospective analysis of systemic steroids with 1 cm representing 1 month (6–8).

Elevated levels of hair cortisol are positively associated with chronic stress (9–12); however, the utility of hair cortisol measurement as a retrospective biomarker of chronic stress has been questioned (13–15). Analyses of hair cortisol in combination with hair cortisone have also been undertaken in other endocrine conditions associated with irregular cortisol levels such as cardiovascular disease (CVD), the metabolic syndrome (MetS) (16), and in cortisol autonomy [adrenocorticotropic hormone (ACTH)-independent cortisol overproduction] in adrenal incidentalomas (17). A study of non-functioning pituitary adenomas showed hair cortisone to be significantly lower in patients with preoperative ACTH deficiency compared to those without ACTH deficiency. Cortisol levels were unchanged, and it was suggested that cortisone in the hair follicle may serve as a preoperative marker for chronic ACTH deficiency (18). Cortisol and cortisone were also shown to be relevant in the diagnosis of overt Cushing’s syndrome (CS), more so than mild CS (19) while cortisone was suggested to be superior to cortisol as a promising biomarker in endogenous CS screening (20). While the diagnosis of CS remains challenging, cyclical CS diagnosis is particularly difficult. The retrospective analysis of hair cortisol and cortisone, the inactive metabolite of cortisol catalyzed by 11β-hydroxysteroid dehydrogenase (11βHSD) type 2, has been suggested to be a useful diagnostic tool for CS, replacing or enhancing current biochemical testing that measures single-point cortisol concentrations.

**Figure 1:**
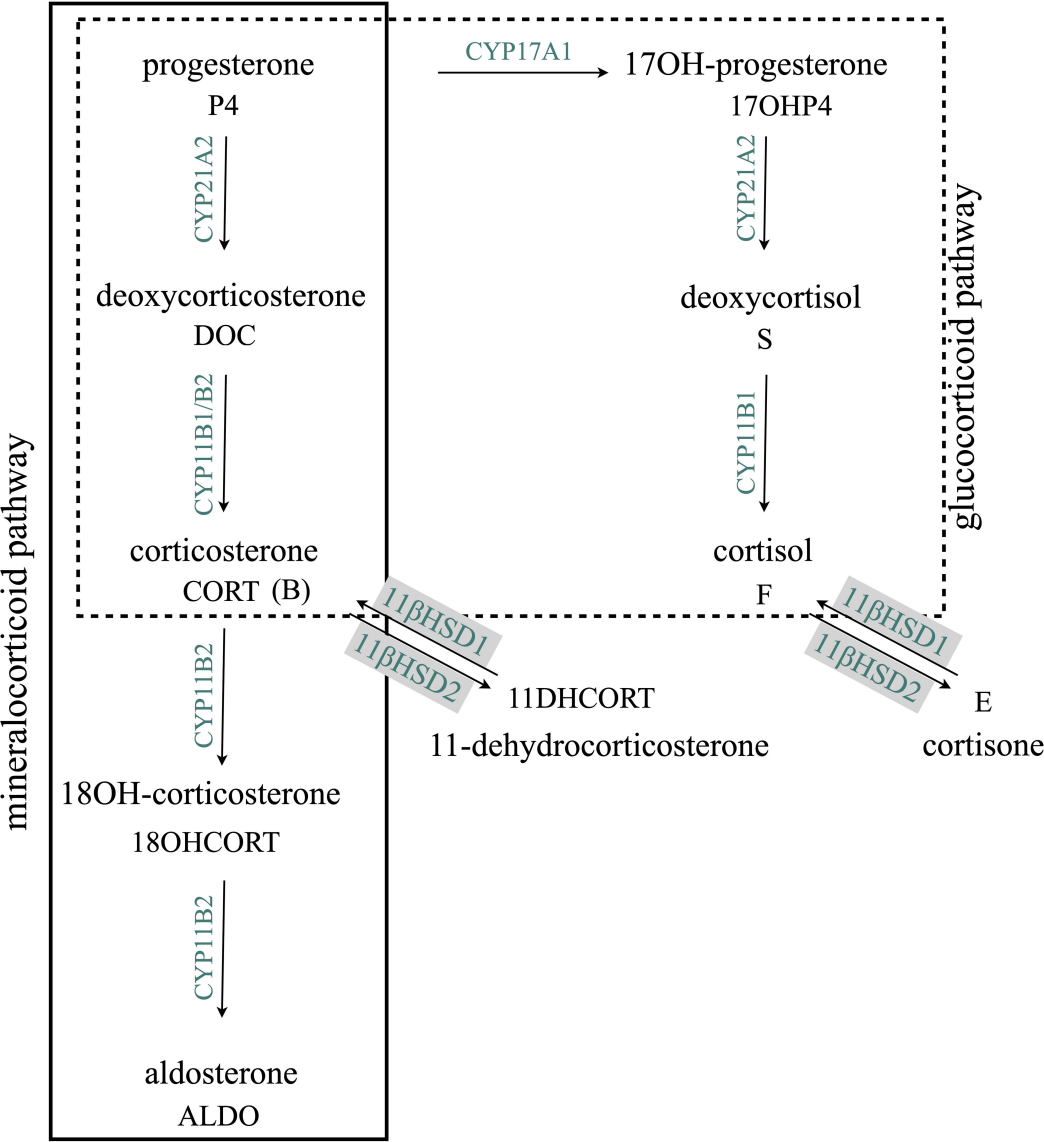
Adrenal mineralocorticoid and glucocorticoid biosynthesis pathways. CYP17A1 is not expressed in the zona glomerulosa; therefore, CYP21A1, expressed throughout the cortex, channels progesterone into the mineralocorticoid pathway. CYP11B2, expressed only in the zona glomerulosa, catalyzes the conversion of deoxycorticosterone to aldosterone. CYP11B1, expressed only in the zona fasciculata and zona reticularis, catalyzes the conversion of deoxycorticosterone to corticosterone and the conversion of deoxycortisol to cortisol. 11βHSD1 and 11βHSD2, expressed in the adrenal and peripheral tissue, catalyzes the interconversion of glucocorticoids, corticosterone, and cortisol to dehydrocorticosterone and cortisone respectively. CYP17A1, cytochrome P45017α-hydroxylase/17-20lyase; CYP21A2, cytochrome P450 21-hydroxylase; CYP11B1, cytochrome P450 11β-hydroxylase; CYP11B2, aldosterone synthase; 11βHSD1 and 11βHSD2, 11β-hydroxysteroid dehydrogenase type 1 and type 2.

Although analyses of adrenal and gonadal steroids are well reported (14, 21–24, 41), investigations of the mineralocorticoid and glucocorticoid pathways have not been a specific focus of hair steroid analysis. In the mineralocorticoid pathway, deoxycorticosterone (DOC) is converted to aldosterone by aldosterone synthase (CYP11B2), shown in **Figure 1**. While the 11β-hydroxylation of DOC to corticosterone (CORT) is catalyzed by both aldosterone synthase (CYP11B2) and cytochrome P450 11β-hydroxylase (CYP11B1), the subsequent hydroxylation at C18 yielding 18-hydroxycorticosterone (18OHCORT) and oxidation yielding aldosterone (ALDO) are only catalyzed by CYP11B2 in the zona glomerulosa. The conversion of deoxycortisol to cortisol in the glucocorticoid pathway is catalyzed by CYP11B1 only. Although certain adrenal glucocorticoid and mineralocorticoid steroids have been investigated in hair (17, 23–25, 41), few have been detected and at very low levels. Glucocorticoids and mineralocorticoids, finely regulated by ACTH and angiotensin II, respectively, within the renin–angiotensin–aldosterone system, have been associated with numerous clinical conditions. Changes in the mineralocorticoid pathway are seen in both conditions of ALDO deficiency and excess as well as primary hyperaldosteronism (PA), one of the most common causes of secondary hypertension. In PA, both circulating CORT and 18OHCORT are elevated together with increased ALDO and decreased renin secretion. In ALDO deficiency, circulating CORT and 18OHCORT are also elevated (26–28). The dysregulation of steroidogenesis is also apparent in the glucocorticoid and mineralocorticoid pathways in congenital adrenal hyperplasia (CAH) subtypes indicated by plasma steroids: in 11β-hydroxylase deficiency (11βOHD), DOC and deoxycortisol are elevated and cortisol and cortisone decreased; in 17α-hydroxylase deficiency (17αOHD) and cytochrome P450 oxidoreductase (PORD), CORT and 18OHCORT are increased and cortisol and cortisone decreased; and in 21-hydroxylase deficiency (21OHD), cortisol and cortisone are decreased (29). Steroid hair analysis in a cohort of untreated Indonesian CAH patients diagnosed with 21OHD and 11βOHD detected elevated androstenedione (A4), 17-hydroxyprogesterone (17OHP4), and testosterone, while cortisol was detected at low levels similar to those found in healthy volunteers (30). Several factors may influence and lead to variability in follicle steroid levels including hair growth rate, cycling phases, hair density, and diameter. It is generally accepted that the average hair growth rate is ~1 cm/month; however, growth rates differ significantly among ethnic groups, 0.82−1.3 cm/month, while in both genders, hair density and diameter vary significantly, 153−233 hair/cm^2^ and 69−89 µm, respectively. Men and women have comparable hair growth rates and percentage of hair in the telogen (rest) phase (7). Studies comparing and correlating steroids in hair and with other matrices are further confounded by the fact that steroids are biosynthesized and metabolized in skin as well as in the hair follicle (31).

Extraction procedures and analytical methodologies determine the detection and accurate quantification of steroids. The analysis of steroids using immunoassays often leads to overestimation of concentrations due to the cross-reactivity of structurally similar hormones potentially skewing steroid ranges (24). Different optimization strategies have been reported; however, the protocols were generally for specific steroids (4, 25, 32, 33). Hair is often not weighed accurately, and while methanol is the general solvent of choice, protocols differ greatly in terms of preparation, extraction temperature, agitation or sonication, single or repeated extractions, and incubation periods. One factor that has remained consistent in reported protocols is the relatively small volume of extraction solvent. Most studies extract 10–50 mg of hair using 0.5–2 mL of methanol followed by, in some instances, solid-phase or liquid–liquid extraction. It is often also assumed that the end tips of long hair are steroid-free when determining matrix effects while others compensate for endogenous steroids by determining absolute and relative matrix effects.

Mineralocorticoids have not been investigated in hair; therefore, this study aimed to analyze a multi-steroid panel of both glucocorticoids and mineralocorticoids present in hair utilizing a novel extraction protocol followed by steroid measurement using a validated ultra-performance convergence chromatography–tandem mass spectrometry (UPC^2^-MS/MS).

## 2 Materials and methods

### 2.1 Materials

Steroids were purchased from Merck (Darmstad, Germany) and Steraloids Inc. (Newport, RI, USA) and included 11-deoxycorticosterone (DOC), corticosterone (CORT), 18-hydroxycorticosterone (18OHCORT), aldosterone (ALDO), deoxycortisol, cortisone, 11-dehydrocorticosterone (11DHCORT), cortisol 21-deoxycortisol (21-dF), dihydrotestosterone (DHT), 11-ketotestosterone (11KT), and 5α-androstan-3α,17β-diol (3αDIOL). Deuterated internal standards were purchased from CDN Isotopes (Quebec, Canada) and Cambridge Isotopes Laboratories (Andover, MA, USA).

CO_2_ and N_2_ were purchased from Afrox (Cape Town, South Africa); methyl tert-butyl ether (MTBE), 2-propanol (99.9%), and formic acid were obtained from Merck (Darmstad, Germany); methanol 215 SpS was purchased from ROMIL Ltd. (Cambridge, England). The Viridis SFC ethylene-bridged hybrid 2-ethylpyridine (BEH 2-EP) column (130 Å, 3.0 × 100 mm, 1.7 μm particle size) and the pre-column ACQUITY UPC2^®^ BEH 2-EP VanGuardTM (2.1 × 5 mm, 1.7 μm particle size) were purchased from Waters Corporation (Milford, USA).

### 2.2 Preparation of steroid and internal standards

Steroid stock solutions, 2 mg/mL, were prepared in absolute ethanol from which master mixes, 1–10,000 ng/mL, containing all the steroids included in the method were prepared in 50% methanol/deionized water. Deuterated-internal standard stock solutions, 1 mg/mL, were prepared in absolute ethanol, diluted to master mixes in 50% methanol. Internal standards, 100 μL, were added to each sample of 50 mg of hair, prior to extractions and contained 1 ng of 21-dF-d8; 5 ng of DHT-d4, 11KT-d3, and cortisol-d4; and 25 ng of 3αDIOL-d3. The internal standard master mix and appropriate dilutions of the steroid standards were used to prepare the reference range of 0.2, 2.0, 10.0, 20.0, 50.0, 100, 200, 1,000, and 2,000 ng/mL in 50% methanol.

### 2.3 Hair collection, preparation, and extraction

#### 2.3.1 Hair collection

Hair was obtained with consent from 15 volunteers (7 women and 8 men) from a local hair salon and the samples were anonymized. Hair designated for steroid analysis was cut as close as possible to the scalp in the lower posterior occipital region, above the nape.

Long hair for the analysis of steroids along the length of the hair follicle was collected from one female and three male clients with long hair (5−8 cm) and four female clients with longer hair (8−12 cm). Hair shaved on the scalp was collected from three men who shaved their heads regularly. One sample of beard hair was collected, and the 1-cm segment shaved on the cheek was used for analysis. Snippet samples from the posterior vertex were from clients with short hair (3–4 cm) having regular haircuts (three women and one man). Samples were stored at −18°C until use.

Hair samples were washed by immersion in isopropanol and agitating on a linear shaker for 5 min after which the samples were dried overnight at 37°C. The dried hair was cut in 1-cm segments, at specific intervals from the scalp ranging from on the scalp to 10.5 cm from the scalp, depending on hair length. The individual segments were subsequently cut into ~1-mm snippets and finely ground in a mortar and pestle with the addition of liquid N_2_. Upon evaporation, the grinding process was repeated twice after the addition of liquid N_2_. The finely ground hair was dried overnight at 37°C and weighed to 50 mg in glass screw cap vials for extraction.

#### 2.3.2 Extraction

Prior to extraction, 2 mL of methanol was added to the samples after which 100 μL of internal standard mix was added. Steroids were extracted using the Thermo Scientific™ ASE system 150, an automated extraction system that minimizes the handling of samples. The parameters on the ASE instrument were set to the following: one static cycle: 5 min static time; 60% rinse volume; 120 s purge time. The solvent used was 100% methanol. The instrument was programmed at 10,342.14 kPa (1,500 psi), conducting three static extractions at 100°C using methanol, followed by purging cycles. Each extraction cycle was carried out with 10 mL of methanol. The methanol extracts from each cycle were pooled in the automated process and subsequently dried under nitrogen at 45°C. Dried residues were resuspended in 175 μL of 50% methanol:deionized water and analyzed using high-throughput UPC^2^-MS/MS.

#### 2.3.3 Preparation of the steroid-free matrix

Snippets cut from leftover ends of long hair (~12–13 cm) were washed, finely cut, and ground as described above. Dried hair, 2.75 g, allowed for an adequate amount of 50-mg samples for extraction as described above using the ASE system for the preparation of a matrix that was free of endogenous steroids. Three samples (two women and one man) were firstly extracted as described above, after which the extracted hair was subjected to a second round of extraction (three static cycles) and prepared for steroid analysis. The dried residues were resuspended in 175 μL of 50% methanol and analyzed. No steroids were detected after the second extraction round, verifying that a single extraction round of three static cycles was sufficient in removing all endogenous steroids. A steroid-free matrix was thus prepared using ~3 g of dried snippets, which were extracted using the ASE system. The methanol extract was discarded, and the extracted hair was dried at 37°C. The dried hair was extracted in batches using the ASE system, after which the methanol extracts were dried as described above.

#### 2.3.4 Determination of matrix effects, limit of detection, lower limit of quantification, recovery, and process efficiency

The dried residue was resuspended in methanol and aliquoted for two blank matrix samples, two zero matrix samples, 20 pre-extraction samples, and 20 post-extraction samples, each sample representing a 50-mg extraction. Matrix-free samples were prepared in 50% methanol with reference standards to include 0.2, 2.0, 10, 20.0, 50, 100, 200, 1,000, and 2,000 ng/mL. Blank matrix samples were prepared to a final volume of 150 μL of 50% methanol without internal standards or reference standards. Zero matrix samples were prepared in a final volume of 150 μL in 50% methanol to which 25 μL of internal standard mix that contained 1 ng of 21-dF-d8; 5 ng of DHT-d4, 11KT-d3, and cortisol-d4; and 25 ng of D3-3αDIOL was added. Post-extraction samples were prepared to a final volume of 150 μL of 50% methanol to which 25 μL of internal standard mix and reference standards (0.2, 2.0, 20, 100, and 200 ng/mL) were added. Four samples were prepared for each of the reference standard concentrations. Pre-extraction samples were prepared as follows: 25 μL of internal standard mix and 30 μL of reference standards (0.2, 2.0, 20, 100, and 200 ng/mL) were added to the resuspended matrix (75 μL) and mixed, after which 370 μL of phosphate buffered saline was added. Four samples were prepared for each of the reference standard concentrations. A liquid/liquid extraction was carried out with 5 mL of MTBE as previously published (48). The dried residue was subsequently resuspended in 50% methanol.

The matrix was determined by comparing the response of the post-extraction samples with matrix-free samples containing equivalent amounts (and concentrations) of the steroids. The matrix effect for each concentration was calculated by dividing the differences in response between these two samples with the response of the matrix-free sample. Recovery was calculated by comparing the response of pre-extraction sample extracts to post-extraction samples. Process efficiency was calculated by comparing the response of post-extraction samples to that of matrix-free samples.

### 2.4 UPC^2^-MS/MS analysis of steroid metabolites

Steroids were analyzed using the UPC^2^-MS/MS system (Waters Corporation, Milford, USA), with a previously validated method (48). Briefly, steroids were chromatographed on a Viridis SFC BEH 2-EP column, injection volume, 2 μL, in a total run time of 15 min per sample. Mass spectrometric detection was carried out on a Xevo TQ-S triple quadrupole mass spectrometer (Waters, Milford, USA) and all steroids were analyzed in multiple reaction monitoring (MRM) mode and positive electrospray ionization mode, with the same previously published mass transitions (**Table 1**).

**Table 1:** Steroid IUPAC and trivial names, abbreviations, and MRM mass transitions.

Steroid Trivial name (abbreviation)	IUPAC name	Mass transitions		
		QN	QL	QL
Deoxycortisol (S)	4-pregnen-17α,21-diol-3,20-dione	247.3 > 123.04	247.3 > 78.94	247.3 > 80.97
Cortisol (F)	4-pregnen-11β,17,21-triol-3,20-dione	363.3 > 121.08	363.3 > 308.98	363.3 > 105.12
Cortisone (E)	4-pregnen-17,21-diol-3,11,20-trione	361.2 > 163.16	361.2 > 120.94	361.2 > 105.19
11-Deoxycorticosterone (DOC)	4-pregnen-21-ol,3,20-dione	331.18 > 159.17	331.18 > 130.89	331.18 > 117.02
Corticosterone (CORT)	4-pregnen-11β,21-diol-3,20-dione	347.2 > 121.1	347.2 > 104.98	347.2 > 97.1
11-Dehydrocorticosterone (11DHCORT)	4-pregnen-21-ol-3,11,20-trione	345.3 > 121.15	345.3 > 163.09	345.3 > 301.2
18-Hydroxycorticosterone (18OHCORT)	4-pregnen-11β,18,21-triol-3,20-dione	363.2 > 269.2	363.2 > 147.0	363.2 > 251.2
Aldosterone (ALDO)	4-pregnen-11β,21-diol-3,18,20-trione	361. 0 > 343.0	361. 0 > 315.0	361. 0 > 97.0

Quantifier, QN; qualifier, QL.

### 2.5 Data processing

Data were collected and processed using Masslynx 4.1 software (Waters Corporation). Steroids were quantified relative to their QN and the assigned labeled internal standard. Steroids were quantified using peak area after applying a smoothing setting of 1/2 to all peak integrations. The internal standard concentration was fixed to 1, and Targetlynx produced standard curves, weighted 1/*x*, excluding the origin so as not to force the line through zero. Statistics were carried out using the GraphPad Prism (version 10.2.3) software (GraphPad Software, San Diego, California). The Shapiro–Wilk test was used to determine normal distribution due to the small number of volunteers and followed by either a nonparametric Mann–Whitney test in cases of data not normally distributed or an unpaired parametric *t*-test (with Welch correction) for normally distributed data.

## 3 Results

A steroid-free matrix was prepared using snippets collected from the ends of long hair. We were unable to obtain a steroid-free matrix extracting hair in methanol having incubated samples for 24 h at 45°C or by Soxhlet extraction using methanol. A matrix was therefore prepared using the ASE system. After the first round of automated extraction, the extracted hair was subjected to a second round of extraction, again using the ASE system. As no steroids were detected, the extracted hair was deemed a suitable steroid-free matrix for determining the limit of detection (LOD), the lower limit of accurate quantification (LLOQ), and the matrix effect.

### 3.1 Calibration range, LOD and LLOQ, and the matrix effect of the steroid metabolites

The LOD and LOQ for all steroids were determined using a concentration range of 0.02 to 2,000 ng/mL (**Table 2**). The standard curves for the analytes included nine points for linear calibration curves with acceptable linearity determined for all curves represented by *R* ^2^ values ranging from 0.9948 to 0.9998. The LOD was determined from the calibration curve in the matrix for each steroid and set at the lowest concentration that yielded a signal-to-noise (S/N) ratio >3 for the quantifier ion. The LLOQ was set at the lowest concentration that yielded a S/N ratio >10 for the quantifier ion and a S/N ratio >3 for the qualifier ion(s). The LODs were 0.2 ng/mL (0.6 pg/mg) and 2.0 ng/mL (6 pg/mg) (**Table 2**). The LLOQs were 2.0 ng/mL (6 pg/mg) for all the steroids except for 11DHCORT, which was 0.2 ng/mL (0.6 pg/mg).

**Table 2:** Limit of detection and lower limit of quantification of steroids.

Steroids	LOD		LLOQ		Calibration range	*R* ^2^
	ng/mL	pg on column	ng/mL	pg on column	ng/mL	
Deoxycortisol	0.20	1.33	2.00	13.33	0.2–2,000	0.9948
Cortisol	0.20	1.33	2.00	13.33	0.2–2,000	0.9959
Cortisone	0.20	1.33	2.00	13.33	0.2–2,000	0.9998
DOC	2.00	13.33	2.00	13.33	0.2–2,000	0.9967
CORT	2.00	13.33	2.00	13.33	0.2–2,000	0.9996
11DHCORT	0.20	1.33	0.20	1.33	0.2–2,000	0.9954
18OHCORT	0.20	1.33	2.00	13.33	0.2–2,000	0.9995
ALDO	2.00	13.33	2.00	13.33	0.2–2,000	0.9989

Linearity (*R* ^2^) of the calibration range of each steroid is shown together with pg steroid on the column representing steroids in a 2-μL injection volume. LOD, limit of detection; LLOQ, lower limit of quantification.

The matrix effect, recovery, and process efficiency are shown in **Table 3**. In the assessment of matrix interference, which would impact the analysis of steroid concentrations in hair, DOC, CORT, and ALDO could not be assessed at the lowest concentration as these were not detected at 0.2 ng/mL. Mixed ionization was observed over the concentration range for all of the steroids except cortisol and DOC. Only ion suppression was observed for cortisol with DOC eliciting only ion enhancement. The matrix effect of other steroids ranged from −17.82% to 0.64% at 0.2 ng/mL, from −17.27% to 4.17% at 2 ng/mL, from −15.76% to 6.96% at 20 ng/mL, from −9.4% to 13.28% at 100 ng/mL, and from −14.73% to 14.69% at 200 ng/mL. Recovery (B) ranged from 86% to 113% at the two lower concentrations and from 86% to 112% at the two high concentrations. At 20 ng/mL, recoveries ranged from 93% to 102%, except for 11DHCORT, which was 123%. The steroid, however, also exhibited high ion suppression, −15.76%. 11DHCORT was the only steroid not detected in all volunteers and was therefore not included in the statistical analyses of steroids in male and female hair samples. Process efficiency showed values ranging from 80% to 108% at the three lower concentrations, and from 82% to 118% at the higher concentrations.

**Table 3:** Validation data for mineralocorticoids and glucocorticoids in the hair follicle.

Steroid	Matrix effect (%)				
	0.2 ng/mL 0.6 pg/mg	2.0 ng/mL 6 pg/mg	20 ng/mL 60 pg/mg	100 ng/mL 300 pg/mg	200 ng/mL 600 pg/mg
Deoxycortisol	−7.54	4.17	5.26	−2.58	3.20
Cortisol	−1.34	−8.39	−2.22	−9.40	−0.77
Cortisone	−17.82	−17.27	1.2	−5.94	14.69
DOC	<LOD	3.52	6.96	6.19	4.76
CORT	<LOD	−3.79	6.30	13.28	10.30
11DHCORT	−2.54	2.25	−15.76	−2.28	−1.07
18OHCORT	0.64	−17.25	−13.74	−0.37	−14.73
ALDO	<LOD	−10.62	0.83	2.98	3.47
Steroid	Recovery (%)				
	0.2 ng/mL 0.6 pg/mg	2.0 ng/mL 6 pg/mg	20 ng/mL 60 pg/mg	100 ng/mL 300 pg/mg	200 ng/mL 600 pg/mg
Deoxycortisol	86.15	98.62	96.36	93.43	88.39
Cortisol	109.37	92.43	96.25	90.08	80.46
Cortisone	102.24	99.36	102.18	102.58	82.42
DOC	<LOD	98.70	94.98	97.28	112.76
CORT	<LOD	105.06	97.00	86.30	83.30
11DHCORT	98.89	97.87	123.13	98.27	88.86
18OHCORT	104.81	106.04	93.16	98.28	96.36
ALDO	<LOD	113.52	102.17	89.27	84.36
Steroid	Process efficiency (%)				
	0.2 ng/mL 0.6 pg/mg	2.0 ng/mL 6 pg/mg	20 ng/mL 60 pg/mg	100 ng/mL 300 pg/mg	200 ng/mL 600 pg/mg
Deoxycortisol	79.66	102.73	101.43	91.02	85.57
Cortisol	107.90	84.68	94.11	81.61	79.84
Cortisone	84.01	82.20	103.41	96.48	94.52
DOC	<LOD	102.16	101.60	103.30	118.13
CORT	<LOD	101.09	103.11	97.76	92.39
11DHCORT	96.39	100.07	103.73	96.03	87.90
18OHCORT	105.48	87.75	80.36	97.93	82.17
ALDO	<LOD	101.46	103.02	91.93	87.30

Data calculated from response values for steroid metabolites ranging from 0.2 to 200 ng/mL, *n* = 4.

### 3.2 Steroid concentrations of female and male hair follicle samples

**Figure 2:**
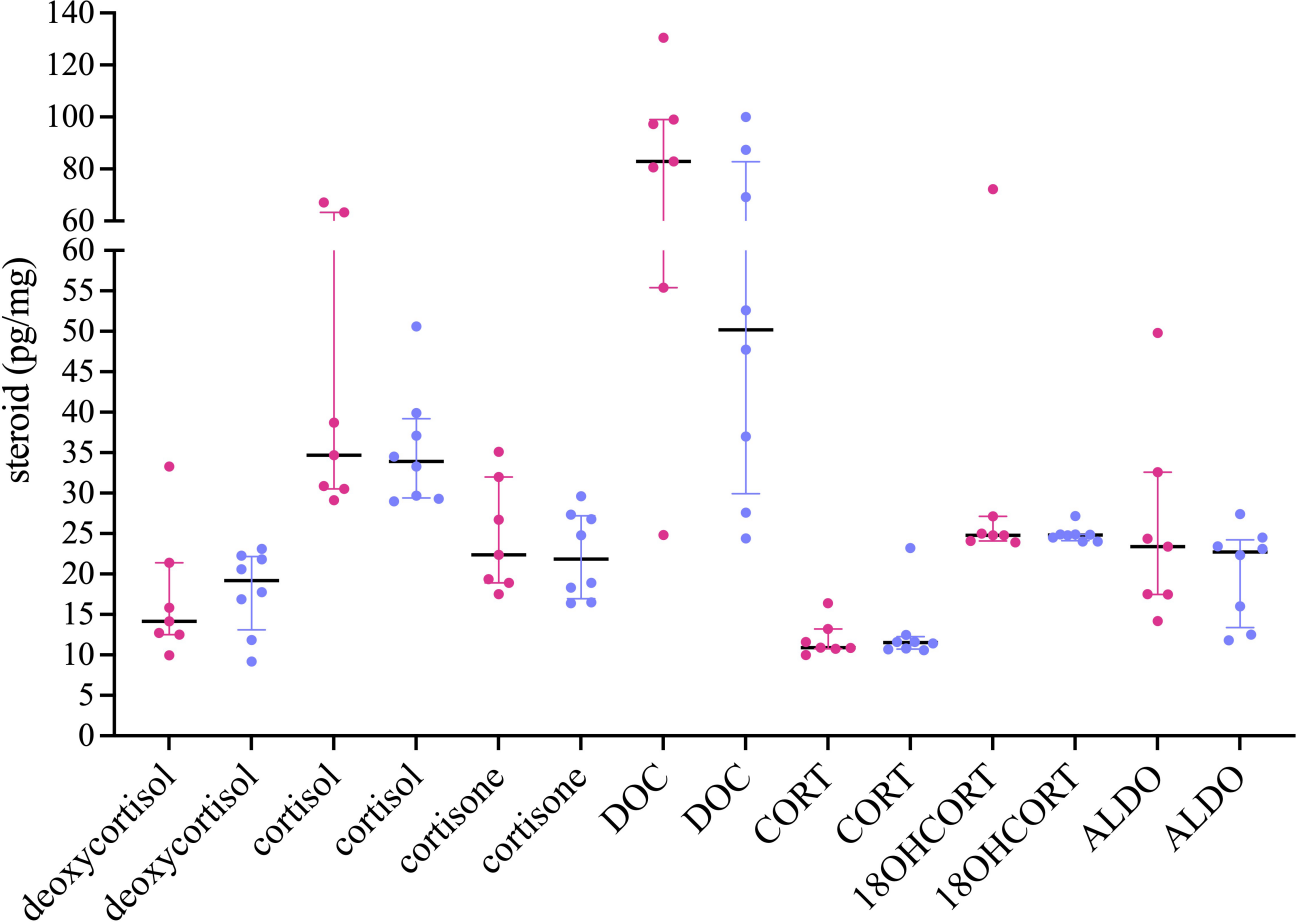
Analysis of glucocorticoid and mineralocorticoid concentrations in hair. Segments, 1 cm were collected from 7 women, 23–48 years and 8 men, 21–53 years with long, medium, and short hair and hair shaved on the scalp. Data are shown as median with interquartile range.

All glucocorticoids and mineralocorticoids were detected above the LLOQ. All the steroids were detected in all the samples except for 11DHCORT. 11DHCORT was detected in one man, 0.92 pg/mg, and in three women, ranging from 0.8 to 11.0 pg/mg. Since there was a degree of fluctuation over the length of the hair follicles, steroid averages were first determined for segments taken from longer hair. Steroid concentrations were compared and neither glucocorticoids nor mineralocorticoids differed significantly in male and female hair samples. DOC was the most abundant steroid in both women and men, with a few exceptions, and showed the greatest inter-individual variation (**Figure 2**).

In one woman (36 years), with lower DOC concentrations (24.83 pg/mg), cortisol (38.70 pg/mg) was detected at a higher concentration. In one man (37 years) with lower DOC concentrations (24.4 pg/mg), cortisol (39.9 pg/mg) was also detected at higher levels. Concentrations were nevertheless comparable to cortisol in other volunteers. Deoxycortisol also showed inter-individual variation in both genders. In men and women, cortisol levels were fairly consistent, showing little inter-individual variation and ranged from 29 to 39 pg/mg, when not including two women and one man with high cortisol concentrations, 61, 63, and 50 pg/mg, respectively.

CORT showed little inter-individual variation in both genders with comparable min/max ranges (**Table 4**). The min/max range of the glucocorticoids, deoxycortisol, cortisol, and cortisone was comparable in men and women showing similar median values. CORT, 18OHCORT, and ALDO were comparable in men and women and showed little inter-individual variation, except for one woman (35 years) with very high 18OHCORT and ALDO, 72.2 and 49.8 pg/mg, respectively. 11DHCORT was also detected at high levels, 11.0 pg/mg.

**Table 4:** Comparison of mineralocorticoid and glucocorticoid concentrations.

Steroid	Women Mean ± SD	Median (Q1, Q3)	Range pg/mg	Men Mean ± SD	Median (Q1, Q3)	Range pg/mg
Deoxycortisol	17.12 ± 7.39	14.2 (12.6, 18.6)	10.0–33.3	17.93 ± 4.77	19.18 (15.6, 21.9)	9.2–23.1
Cortisol	42.05 ± 14.97	34.70 (30.7, 51.0)	29.1–67.1	35.42 ± 6.80	33.91 (29.6, 37.8)	29.0–50.6
Cortisone	24.56 ± 6.38	22.38 (19.1, 29.4)	17.5–35.2	22.33 ± 5.01	21.85 (17.9, 26.9)	16.4–29.6
DOC	81.51 ± 31.28	82.96 (68.0, 98.1)	24.8–130.5	55.74 ± 25.84	50.18 (34.6, 73.7)	24.4–100
CORT	11.96 ± 2.03	10.90 (10.8, 12.4)	10.0–16.4	12.80 ± 3.97	11.52 (10.8, 11.8)	10.6–23.2
11DHCORT*	6.09 ± 3.72	5.26 (3.6, 8.1)	2.0–11.0	—	—	nd–0.9
18OHCORT	31.70 ± 16.56	24.80 (24.5, 26.1)	23.9–72.2	24.89 ± 0.93	24.83 (24.4, 24.9)	24.0–27.2
ALDO	25.61 ± 11.36	23.38 (17.5, 28.5)	14.2–49.8	20.13 ± 5.49	22.72 (15.1, 23.7)	11.8–27.4

*Women, *n* = 3; and men, *n* = 1.

Steroid concentrations (pg/mg) in women (*n* = 7) and men (*n* = 8) in segments taken from long, medium, and short hair. Steroids not detected = nd. Data are shown as mean ± SD, median and range.

Since many studies report the analysis of steroids only in segments close to the scalp, we analyzed steroid concentrations in these segments in women and men, in shaved scalp hair segments in men, and in one shaved cheek segment (**Table 5A**). Data also showed that DOC and deoxycortisol exhibited the greatest inter-individual differences in both women and men. DOC was also the most abundant steroid in these segments in most samples spanning wide ranges and was comparable in women and men ranging from 27 to 117 pg/mg and from 30 to 136 pg/mg, respectively (**Table 5B**). Cortisone and cortisol did not show inter-individual variation, with comparable median values for both steroids in men and women. CORT levels were comparable in men and women apart from one man with CORT levels ~2.6-fold higher. 18OHCORT showed no inter-individual variation with the same median values in men and women (24.8 pg/mg). Little inter-individual variation was detected in the ALDO concentrations with comparable male and female min/max ranges. None of the glucocorticoids or mineralocorticoids differed significantly, with means indicating DOC being the most prominent steroid on the scalp, although this was not the case in all samples with cortisol and cortisone higher in one woman and cortisol higher in one man.

**Table 5A:** UPC^2^-LC-MS/MS analysis of mineralocorticoids and glucocorticoids close to skin.

Females Steroid	26 years	36 years	36 years	38 years	48 years
	Segment cut on scalp				
Deoxycortisol	19.1	7.5	21.4	29.7	18.5
Cortisol	32.4	42.0	34.7	31.3	32.6
Cortisone	17.0	31.4	32.0	18.9	22.9
DOC	90.4	27.2	55.4	117.3	90.4
CORT	11.4	10.1	10.0	13.1	10.5
11DHCORT	1.3	nd	nd	nd	nd
18OHCORT	34.9	23.8	24.8	24.3	24.9
ALDO	18.1	12.9	14.2	26.5	23.9

Males Steroid	21 years	24 years	28 years	48 years	53 years	49 years	30 years	28 years Beard
	Segment cut on scalp				Shaved on skin			
Deoxycortisol	26	16.9	18.9	16.6	9.2	21.8	20.6	7.2
Cortisol	30.5	28.2	29.3	36.7	33.3	37.1	50.6	33.3
Cortisone	16.9	16.2	28.4	27.6	18.9	29.6	16.4	24.3
DOC	66.4	136.6	58.4	30.7	69.2	37.0	52.6	37.1
CORT	11.9	11.6	34.7	12.4	10.6	10.7	10.8	14.7
11DHCORT	nd	nd	nd	0.9	nd	nd	nd	nd
18OHCORT	26.2	25.4	26.6	23.9	24.8	24.9	24.9	27.4
ALDO	23.2	20.8	26.2	28.0	11.8	27.4	23.1	36.4

Steroid concentrations (pg/mg) in segments cut on scalp and in segments shaved on the scalp and cheek. Steroids not detected, nd.

**Table 5B:** Comparison of mineralocorticoids and glucocorticoids.

Steroid	Women	Median (Q1, Q3)	Range pg/mg	Men	Median (Q1, Q3)	Range pg/mg
	Mean ± SD (*n* = 5)			Mean ± SD (*n* = 7)		
Deoxycortisol	19.64 ± 7.3	19.1 (18.5, 21.4)	7.5–29.7	17.15 ± 5.9	18.9 (16.8, 21.2)	9.2–26.0
Cortisol	35.48 ± 4.3	32.6 (32.4, 34.7)	31.3–42.0	34.89 ± 6.7	33.3 (29.9, 36.9)	28.2–50.6
Cortisone	25.48 ± 7.6	22.9 (18.9, 31.4)	17.0–32.0	22.29 ± 5.4	18.9 (16.7, 28.0)	16.2–29.6
DOC	74.94 ± 32.3	90.4 (55.4, 90.4)	27.2–117.3	61.0 ± 31.5	58.4 (44.8, 67.8)	30.7–136.6
CORT	11.67 ± 1.3	10.5 (10.1, 11.4)	10.0–13.1	14.67 ± 7.7	11.6 (10.8, 12.2)	10.6–34.7
18OHCORT	27.38 ± 4.2	24.8 (24.3, 24.9)	23.8–34.9	25.52 ± 1.1	24.9 24.9, 25.8	23.9–26.6
ALDO	25.44 ± 11.2	18.1 (14.2, 23.9)	12.9–26.5	24.61 ± 6.6	23.2 22.0, 26.8	11.8–28.0

Steroid concentrations (pg/mg) in segments cut on the scalp and shaved on the scalp. Data are shown as mean ± SD, median and range.

Analysis of the beard sample from the 28-year-old male volunteer showed that all glucocorticoids and mineralocorticoids were detected in the sample except for 11DHCORT. Cortisol, cortisone, and 18OHCORT concentrations in the beard sample were comparable to concentrations on the scalp of the same volunteer. Deoxycortisol, DOC, and CORT were 2.6-, 1.6-, and 2.4-fold lower, respectively, and ALDO was 1.4-fold higher in the beard sample than in the corresponding hair sample.

Since it is generally accepted that hair grows 1 cm per month, we analyzed samples in 1-cm segments cut along the length of the hair follicle. These were designated according to their sampling distance from the scalp at 0.5, 1.5, 3.5, 6.5, and 10.5 cm, depending on hair length. The segments represent periods of 1, 2, 4, 7, and 11 months (**Table 6A**). DOC and deoxycortisol fluctuated the most over the length of the hair follicle in long (A), medium (B), and short (C) hair. 11DHCORT was detected in only one woman over the length of the hair follicle (A) and in two medium hair samples (B).

**Table 6A:** UPC^2^-LC-MS/MS analysis of mineralocorticoids and glucocorticoids along the hair follicle.

(i) Steroid	Female, 26 years					Female, 38 years				
	Growth period (months)									
	1	2	4	7	11	1	2	4	7	11
Deoxycortisol	19.1	10	6.9	14.3	20.5	29.7	47.2	41.3	27.0	21.2
Cortisol	32.4	29.6	27.9	27.7	28.0	31.3	33	30.0	28.8	29.6
Cortisone	17	17.7	18.3	17.1	17.4	18.9	20.9	18.0	19.5	19.5
DOC	90.4	75.2	75.9	96.5	76.8	117.3	121.7	94.7	65.0	87.6
CORT	11.4	10.8	10.7	9.9	11.6	13.1	14.9	13.6	12.9	11.5
11DHCORT	1.3	0.8	9	5	10.2	nd	nd	nd	nd	nd
18OHCORT	34.9	25.3	27.4	24	24	24.3	24.3	25.3	24.2	25.9
ALDO	18.1	18.6	18.7	13.7	18.2	26.5	31.2	20.8	18.2	20.2
(ii) Steroid	Female, 48 years				Male, 48 years					
	Growth period (months)									
	1	2	4	7	1	2	4	7		
Deoxycortisol	18.5	20.3	15.8	8.7	16.6	14	9.9	6.8		
Cortisol	32.6	30.9	33.1	26.9	36.7	34.9	33.9	32.6		
Cortisone	22.9	23.1	24.4	19.1	27.6	27.3	25.9	26.3		
DOC	90.4	82.7	87.4	61.9	30.7	25.1	32.5	22.0		
CORT	10.5	11.3	11.2	10.6	12.4	12.8	12.3	12.3		
11DHCORT	nd	2.1	nd	1.9	0.92	nd	nd	nd		
18OHCORT	24.9	25.0	25.6	24.5	23.9	25.6	24.2	24.3		
ALDO	23.9	26.2	28.6	18.7	28	21.4	21.5	22.8		
(iii) Steroid	Female, 36 years			Male, 21 years			Male, 24 years			
	Growth period (months)									
	1	2	4	1	2	4	1	2	4	
Deoxycortisol	7.5	6.9	15.5	26	21.3	19.5	16.9	18.7	15	
Cortisol	42	41.6	32.5	30.5	28.7	29.8	28.2	28.6	30.5	
Cortisone	31.4	24.6	24.1	16.9	17	21.1	16.2	15.8	18.8	
DOC	27.2	32.6	14.7	66.4	104.9	90.8	136.6	52.3	113.8	
CORT	10.1	10.1	12.1	11.9	11.1	11.3	11.6	11.2	11.7	
18OHCORT	23.8	23.8	24.7	26.2	24.5	23.9	25.4	24.1	24.4	
ALDO	12.9	12.1	27.5	23.2	20.1	23.7	20.8	19.8	11.7	

Steroid concentrations (pg/mg) in 1-cm segments in (i) long hair 1–11 months, (ii) medium-length hair 1–7 months, and (iii) short hair 1–4 months. Steroids not detected, nd.

**Table 6B:** Glucocorticoid and mineralocorticoid concentrations in hair follicles spanning 1 to 11 months’ growth.

Steroid	Mean ± SD *n* = 7	Median (Q1, Q3)	Range pg/mg
Deoxycortisol	18.34 ± 9.73	16.9 (12.0, 20.9)	6.8–47.2
Cortisol	31.57 ± 3.80	30.5 (28.8, 32.8)	26.9–42.0
Cortisone	20.99 ± 4.19	19.5 (17.6, 24.3)	15.8–31.4
DOC	73.08 ± 33.78	76.8 (42.5, 92.8)	14.7–136.6
CORT	11.66 ± 1.14	11.5 (11.0, 12.3)	9.9–14.9
18OHCORT	25.13 ± 2.13	24.5 (24.2, 25.4)	23.8–34.9
ALDO	21.00 ± 5.05	20.8 (18.4, 23.8)	11.7–31.2

Data represent four female and three male volunteers. Steroid concentrations (pg/mg) in segments from long and medium-length hair. Data are shown as mean ± SD, median and minimum and maximum range.

Cortisol and cortisone did not fluctuate in any of the segments along the follicles of either the male samples or female samples as is reflected in concentrations remaining close to the median of 31.1 and 19.5 pg/mg (**Table 6B**). Analysis of hair segments of the 48-year-old male volunteer (B) showed that both cortisol and cortisone were at the higher end of the min/max range.

CORT was comparable in both genders with concentrations not varying along the length follicle. Neither 18OHCORT nor ALDO fluctuated greatly in long hair (i) except for one woman (38 years) with ALDO concentrations ranging from 18 to 31 pg/mg. Fluctuation over the hair follicle contributed to ALDO ranges being wider than 18OHCORT.

The analysis of medium-length hair, 5 months’ growth (ii) of one woman and two men again showed that DOC fluctuated along the length of the follicle in both genders (**Table 6A**), more so in the male samples, together with inter-individual variance confirming wide ranges (**Table 6B**). Inter-individual variation and fluctuations in deoxycortisol concentrations are evident; however, the fluctuation was not observed in the 21-year-old man (iii). CORT and 18OHCORT followed the same unvarying trend with no inter-individual variation measured over the different lengths of the hair follicle, also reflected in narrow ranges. ALDO fluctuated markedly over the hair follicle of some of the volunteers, reflected in the wide range of 11.7–31.2 pg/mg and also showed inter-individual variation.

## 4 Discussion

This is the first study to identify and quantify a multi-steroid panel in hair and a beard sample representing the adrenal profile of mineralocorticoids and glucocorticoids. We employed an automated extraction system that ensured no residual steroids remained after a single round of triplicate extractions, with greater volumes of methanol together with higher temperatures. The novel extraction approach of this study enabled the detection of steroids previously not detected in other investigations.

We were able to show that some steroids remain consistent over the length of the hair follicle and that fluctuations and inter-individual variance of steroid concentrations also contribute to the wider steroid ranges.

Glucocorticoids and mineralocorticoids in both men and women did not differ. For the first time, we report that within the glucocorticoid pathway, deoxycortisol was detected in men, which has been investigated but not detected in male-pattern baldness, adrenal incidentalomas, and nonfunctioning pituitary adenoma (17, 18, 21). Deoxycortisol showed marked inter-individual variance of concentrations, detected at comparable levels in male and female samples ranging from 9.2 to 33.3 pg/mg, and 30- to 100-fold higher than previously reported in women. Deoxycortisol was detected at a 19% frequency in a cohort of 196 healthy women ranging from 0 to 0.33 pg/mg in hair (24). Low deoxycortisol levels have also been reported in hair from newborns with comparable levels in both genders, 5.8–6 pg/mg (34) while endogenous deoxycortisol was not previously detected in men or women (23). Establishing normal ranges would be difficult given the varying concentrations published to date. Cortisol, extracted from 50 mg, ranged from 0.12 to 87 pg/mg (4, 24, 35–37), and that extracted from 20 mg ranged from 0.8 to 60 pg/mg (3, 38–41). Our cortisol levels in men and women were comparable, ranging from 28 to 42 pg/mg with little inter-individual variance when considering cortisol of three volunteers as outliers ranging from 50 to 67 pg/mg. Wide ranges have also been reported for cortisone, 5.6–148.8 pg/mg extracted from 20 mg (3, 38–40) and 0.58–202 pg/mg extracted from 50 mg of hair (4, 24, 35–37, 41). Our data did not show inter-individual variation of cortisone concentrations in either men or women and ranged from 16.4 to 35.1 pg/mg. In all hair samples analyzed, cortisol was always higher than cortisone, ~1.65-fold, in accord with cortisol circulating at fivefold higher concentrations than cortisone. The converse is reported in the literature with cortisone concentrations higher than cortisol in hair. This discrepancy is likely due to our extraction protocol favoring the efficient extraction of both steroids. The interconversion of cortisol and cortisone upon diffusion into the follicle is possible as both 11βHSD type 1 and type 2 are expressed in keratinocytes and in the pilosebaceous unit (31, 42–44), potentially favoring the conversion of cortisone to cortisol upon diffusion into the hair follicle. The presence of steroid-5α-reductase and 3α-hydroxysteroid dehydrogenase in the hair follicle (45) may metabolize cortisol and cortisone, thus influencing glucocorticoid levels.

Few studies have investigated cortisol and cortisone in hair in the context of specific clinical conditions. Hair glucocorticoids in CS have been of interest for some time, although studies have not been consistent regarding cortisol and cortisone levels. In an investigation into the diagnosis of overt and mild CS, both hair cortisol and cortisone were higher in patients than in controls. It was concluded that both glucocorticoids had more utility for the diagnosis of overt CS than for mild CS (19). Cortisol and cortisone in control subjects were lower than in our female volunteers, 4- and 1.6-fold, respectively. Levels that were lower than our measured levels have also been reported for both controls and endogenous CS patients: hair cortisol and cortisone levels in healthy controls were 2.7 and 8.2 pg/mg, respectfully, and both were higher in patients (4.7- and 4.9-fold, respectively). It was nevertheless concluded that hair cortisone was more accurate than hair cortisol in screening for endogenous CS (20). A more recent study investigating cortisol autonomy in patients with adrenal incidentalomas detected comparable hair cortisol and cortisone levels in patients and controls: cortisol, 2.7 and 3.4 pg/mg, and cortisone, 10.5 and 13.2 pg/mg. Deoxycortisol was not detected in controls or patients, and the authors concluded that hair glucocorticoids were unsuitable for diagnostic purposes (17). The discrepancy to the findings we report is likely technical due to the small quantity of hair and small solvent volumes.

In the mineralocorticoid pathway, DOC was the most prominent steroid of the adrenal panel of both genders and 1.5-fold higher in female samples, but not significantly so. Inter-individual variation in concentrations was observed in both genders potentially contributing to the wider min/max ranges. DOC has not been widely reported with our study detecting the steroid for the first time in men ranging from 22 to 136.6 pg/mg. DOC levels have been reported in healthy women ranging from undetected to 0.69 pg/mg, with a detection frequency of 87/167 (24), while it was not detected in other studies (21, 23). DOC was markedly higher in our study ranging from 15 to 130 pg/mg. We observed that cortisol and cortisone were higher when DOC concentrations were at the minimum range.

Although CORT has been investigated and not detected by other groups (18, 23, 25), low levels have been reported in men (0.46–2.39 pg/mg) and women ranging from 0 to 2.4 pg/mg (24, 38). In a more recent study, CORT was detected at higher concentrations in men and women, 3.5–7.6 and 5.2–11.2 pg/mg, respectively (41). We detected similar concentrations that were also comparable in men and women, 11.5 and 10.9 pg/mg, respectively, with higher CORT in two volunteers (23.2 and 16 pg/mg). We detected 11DHCORT, the 11βHSD2 product of CORT, in only four volunteers (0.9–11 pg/mg) while it has been reported to range from 0.33 to 3.7 pg/mg in women and from 1.4 to 3.6 pg/mg in men. 11DHCORT, together with cortisol and cortisone, was also detected at comparable levels in the beard (4). Although cortisol and cortisone concentrations were higher in our beard sample, they were also comparable to levels in the associated hair segment cut close to the scalp. We did not detect 11DHCORT in the beard sample. Other steroid concentrations in the beard differed considerably; deoxycortisol, DOC, and CORT were higher in hair while ALDO was 1.4-fold higher in the beard.

Our study is the first to analyze 18OHCORT in hair and detect comparable concentrations in both genders except for one female volunteer (age 35) who also had high CORT levels. Both 18OHCORT (72.2 pg/mg) and ALDO (49.8 pg/mg) were markedly higher than in other volunteers. Interestingly, high levels of 11DHCORT were also detected in this volunteer. The volunteer may have an underlying condition of which she is unaware. Excluding the volunteer, 18OHCORT did not show inter-individual variation and ranged from 23.9 to 35 pg/mg with a median of 24.8 pg/mg in both men and women. Although the ALDO median for men and women was 22.7 and 23.4 pg/mg, respectively, ALDO showed a degree of inter-individual variation and fluctuation. It was recently suggested that urinary 18OHCORT, together with ALDO and tetrahydroaldosterone, may serve as new biomarkers for the diagnosis of PA as these were shown to be 2.5-, 1.75-, and 2-fold higher, respectively, in patients diagnosed with PA (28). Previous investigations into PA also suggested 18OHCORT as a marker of PA. Elevated circulating 18OHCORT and ALDO levels have been detected in PA patients due to an adenoma or hyperplasia, although 18OHCORT and ALDO levels were affected by patient posture (26). Both 18OHCORT and 18-hydroxydeoxycorticosterone (18OH-DOC) have been included in investigations into hypoaldosteronism. All mineralocorticoid pathway steroids in circulation were analyzed to determine utility in the differential diagnosis of malignant from non-malignant tumors, and data suggested that the pathway was not disrupted in non-malignant tumors (46). Both 18OHCORT and 18OH-DOC were also subsequently shown to be elevated in circulation in neonates diagnosed with aldosterone synthase deficiency (27).

ALDO in hair has not been reported in men and has only recently been reported in healthy women, but at a very low frequency, 14/196, ranging from 0 to 0.47 pg/mg (24). We detected ALDO ~50-fold higher and at comparable concentrations in men and women, 22.7 and 23.4 pg/mg, respectively. Circulating ALDO is ~1,000 times lower than cortisol (47), a relationship that is not reflected in our ALDO or cortisol hair follicle concentrations. To date, ALDO has not been detected in the skin. Data suggest that either ALDO in the hair follicle is taken up from capillaries or aldosterone synthase (CYP11B2) may be expressed in the follicle environment converting CORT to ALDO in the mineralocorticoid pathway as we also detected 18OHCORT. Hair ALDO, with the inclusion of its precursors, may be a better diagnostic marker for hyperaldosteronism with utility in the interpretation of the aldosterone–renin ratio.

Since most hair analysis is generally carried out with samples taken close to the scalp, we also compared segments cut only on the scalp of women and men to segments shaved on the scalp. Analysis showed the same trend with DOC generally being the most abundant steroid and, together with deoxycortisol, showing the most inter-individual variance. In those volunteers with lower DOC levels, cortisol concentrations were higher, but within the min/max range, suggesting a potential association that may be evident in larger cohorts. Analysis of segments cut on the scalp only allows the assessment of inter-individual variance that may contribute to wide ranges while concentrations over the length of long hair (7 and 11 months′ growth) allow the assessment of fluctuations in steroid levels in individuals, possibly related to physiological or endocrinological events. Segmental analysis of long hair also enabled a better assessment of inter-individual variations compared to single segments taken at the scalp. Inter-individual variations were confirmed for DOC and deoxycortisol. In the glucocorticoid pathway, deoxycortisol varied markedly along the follicle, while cortisol and cortisone remained consistent in both genders. Assessment of steroids in the mineralocorticoid pathway over the length of the hair follicle indicates that CORT, 18OHCORT, and ALDO may be ideal candidates in the diagnosis of PA and aldosterone synthase deficiency as these steroids did not vary along the hair follicle.

Limitations of this study are the small number of volunteers and the limited samples that were analyzed in such depth. The inter-individual variance, the fluctuation, and the uniform steroid concentrations observed along the length of the hair follicle, as well as our detection of higher cortisol concentrations compared to cortisone, need to be validated in a larger cohort of subjects and samples using ASE protocols described in this study. It should, however, be noted that steroids detected in the hair follicle may not all be of adrenal origin. The dermis, being highly vascularized, enables diffusion of steroids, and the uptake of steroids into the follicle has not been conclusively established in humans (14). In addition, skin cells express all the steroidogenic enzymes required for the biosynthesis of glucocorticoids, androgens, and estrogens, *de novo* from cholesterol or from circulating systemic precursors, regulated by corticotropin-releasing hormone (CRH) and ACTH (31) and may also contribute to follicle steroids.

## 5 Conclusion

Through a superior steroid extraction procedure and state-of-the-art steroid measurement, this study is the first to have identified and quantified all glucocorticoids and mineralocorticoids over the length of the follicle representative of the adrenal multi-steroid panel. We detected, for the first time, 18OHCORT in men and women, and deoxycortisol, DOC, and ALDO in men. All steroids were detected above the LLOQ, except for 11DHCORT with a detection frequency of 100%. In women, deoxycortisol, ALDO, and DOC were 43, 50, and 120 times higher than previously reported, respectively. The levels of cortisol, cortisone, 11DHCORT, and CORT were in line with previous reports, but with less inter-individual variance. In both genders, precursors deoxycortisol in the glucocorticoid pathway and DOC in the mineralocorticoid pathway fluctuated considerably along the hair follicle while cortisol and cortisone did not fluctuate. In the mineralocorticoid pathway, CORT, 18OHCORT, and ALDO levels did not differ in women or men, suggesting that these three steroids in the hair follicle may be the new biomarkers for clinical conditions associated with hyper- and hypoaldosteronism. The hair follicle provides an ideal matrix circumventing the consequence of circadian rhythm as well as patient position or posture.

## Data Availability

The original contributions presented in the study are included in the article/supplementary material. Further inquiries can be directed to the corresponding author.
